# *Cymbopleuranatellia* – a new species from Transbaikal area (Russia, Siberia) described on the basis of molecular and morphological investigation

**DOI:** 10.3897/phytokeys.183.72285

**Published:** 2021-10-19

**Authors:** Anton Glushchenko, Evgeniy Gusev, Yevhen Maltsev, John Patrick Kociolek, Irina Kuznetsova, Maxim Kulikovskiy

**Affiliations:** 1 К.A. Timiryazev Institute of Plant Physiology RAS, IPP RAS, 35 Botanicheskaya St., Moscow, 127276, Russia К.А. Timiryazev Institute of Plant Physiology RAS, IPP RAS Moscow Russia; 2 University of Colorado, Boulder; University of Colorado Museum of Natural History, Boulder, Colorado, 80309, USA University of Colorado, Boulder Boulder United States of America; 3 Department of Ecology and Evolutionary Biology, University of Colorado, Boulder, Colorado, 80309, USA University of Colorado Museum of Natural History Boulder United States of America

**Keywords:** Bacillariophyceae, *Cymbopleura*, morphology, molecular investigation, new species, Russia

## Abstract

A new cymbelloid diatom species from the genus *Cymbopleura* (Krammer) Krammer is described on the basis of molecular and morphological investigations. *Cymbopleuranatellia* Glushchenko, Kulikovskiy & Kociolek, **sp. nov.** is, on the basis of results with molecular data, close to *C.naviculiformis* (Auerswald ex Heiberg) Krammer. The two species differ both by molecular distance and morphological features. Morphologically, *C.natellia***sp. nov.** is compared with several other species in the genus. This work is a pioneer investigation of cymbelloid taxa using molecular tool from Transbaikal area.

## Introduction

Cymbelloid diatoms have unusually high diversity in ancient Lake Baikal but we almost have no information about them in waterbodies surrounding this lake. Historically, cymbelloid diatoms have been referred to taxa with dorsiventral symmetry (Round et al. 1990; Kulikovskiy et al. 2016). However, results from molecular investigations and a better understanding of morphological features have shown that genera with naviculoid symmetry also belong to this monophyletic group. New diatom genera from Lake Baikal, such as *Ochigma* Kulikovskiy, Lange-Bertalot & Metzeltin (Kulikovskiy et al. 2012), *Paraplaconeis* Kulikovskiy, Lange-Bertalot & Metzeltin (Kulikovskiy et al. 2012) and *Khursevichia* Kulikovskiy, Lange-Bertalot & Metzeltin (Kulikovskiy et al. 2012) are good examples of naviculoid diatoms that are more closely related to cymbelloid taxa (Kulikovskiy et al. 2012, 2016). Lake Baikal includes many “typical” dorsiventral genera and species that were comprehensively investigated by Kulikovskiy et al. (2012). In that work we described many new species from the genera *Cymbella* C.A. Agardh, *Encyonema* Kützing and *Cymbopleura* (Krammer) Krammer (Kulikovskiy et al. 2012) based on valve morphology. However, these new taxa were described without molecular investigation. Our understanding of the diversity of algae in ancient lake systems will also be enhanced by using molecular methods.

Members of the genus *Cymbopleura* were previously considered to be members of the genus *Cymbella*. *Cymbopleura* was separated from *Cymbella* based on the features of absence of apical pore fields and central raphe ends turned towards the ventral side of valve (Krammer 1982; Kulikovskiy et al. 2012). The new genus *Karthickia* Kociolek, Glushchenko & Kulikovskiy is very similar to *Cymbopleura* except that it differs by the presence of a single external stigma opening that has two elongated slit-like openings internally, residing within two longer central striae (Glushchenko et al. 2019). This new genus was described from Southeast Asia. Another new genus that is similar to *Cymbopleura* is *Yasnitskya* Rodionova & Pomazkina (Pomazkina and Rodionova 2014). This genus is characterized by interesting internal areolar morphology. *Yasnitskya* was also described on the basis morphological investigation only.

The aim of the present report is to begin a series of studies using molecular methods to study the identity and systematic placement of cymbelloid diatoms from Lake Baikal and surrounding watrebodies, with the description of new species from the genus *Cymbopleura*. We plan that this work as our pioneer investigation of dorsiventral species from this area and that this work will be continued in future with new molecular data for other known taxa in this lineage.

## Materials and methods

### Sample collection

The sample used in the present report was collected from Eastern Siberia, Buryatia by E.S. Gusev and M.S. Kulikovskiy on 15.07.2011. It was designated as No. 11.2 and was collected from the Zagza River, periphyton, scraping from macrophytes, t = 14 °C, pH = 8.5, conductivity = 40 μS cm^-1^, 52°31.656'N, 107°05.114'E.

### Culturing

A subsample of each collection was added to WC liquid medium (Guillard and Lorenzen 1972). A monoclonal strain was established by micropipetting a single cell under an inverted microscope. Non-axenic unialgal cultures were maintained in WC liquid medium at 22–25 °C in a growth chamber with a 12:12 h light:dark photoperiod. The strain investigated here was designated B209.

### Preparation of slides and microscope investigation

The culture was treated with 10% hydrochloric acid to remove carbonates and washed several times with deionized water for 12 h. Afterwards, the sample was boiled in concentrated hydrogen peroxide (≈ 37%) to remove organic matter. It was washed again with deionized water four times at 12 h intervals. After decanting and filling with deionized water up to 100 ml, the suspension was pipetted onto coverslips and left to dry at room temperature. Permanent diatom preparations were mounted in Naphrax. Light microscopic (LM) observations were performed with a Zeiss Axio Scope A1 microscope equipped with an oil immersion objective (× 100, n.a. 1.4, differential interference contrast [DIC]) and Axiocam ERc 5s camera (Zeiss). Valve ultrastructure was examined by means of scanning electron microscopes JEOL JSM-6510LV (JEOL Ltd., Japan) operating at 15 kV and 8 mm of working distance (IBIW, Institute for Biology of Inland Waters RAS, Borok, Russia). For scanning electron microscopy (SEM), part of the suspensions was fixed on aluminum stubs after air-drying. The stubs were sputter-coated with 50 nm of Au by means of a Eiko IB 3 (Eiko Engineering, Japan).

The cleaned material, sample and slide are deposited in the collection of MHA, Main Botanical Garden Russian Academy of Science, Moscow, Russia. The type slide was designated B209.

### Molecular investigation

Total DNA from the studied strain was extracted using Chelex 100 Chelating Resin, molecular biology grade (Bio-Rad Laboratories, USA), according to the manufacturer’s protocol 2.2. Partial 18S rDNA (435 bp, including the highly variable V4 region) gene was amplified using primers D512for and D978rev from Zimmermann et al. (2011).

Amplification was carried out using premade polymerase chain reaction (PCR) mastermixes (ScreenMix by Evrogen, Russia). Amplification conditions for 18S rDNA gene were as follows: initial denaturation for 5 min at 95 °C followed by 35 cycles of 30 s denaturation at 94 °C, 30 s annealing at 52 °C, and 50 s extension at 72 °C, with the final extension for 10 min at 72 °C. PCR products were visualized by horizontal electrophoresis in 1.0% agarose gel stained with SYBRTM Safe (Life Technologies, USA). The products were purified with a mixture of FastAP, 10×FastAP Buffer, Exonuclease I (Thermo Fisher Scientific, USA), and water. The sequencing was performed using a Genetic Analyzer 3500 instrument (Applied Biosystems, USA).

Editing and assembling of the consensus sequences were carried out by processing the direct and reverse chromatograms in Ridom TraceEdit (ver. 1.1.0) and Mega7 software (Kumar et al. 2016). The reads were included in the alignments along with corresponding sequences of 74 diatom species downloaded from GenBank (taxa names and Accession Numbers are given in Fig. 5). Five diatom species from Rhopalodiaceae were chosen as the outgroups.

The nucleotide sequences of the 18S rDNA gene were aligned separately using the Mafft v7 software and the E-INS-i model (Katoh and Toh 2010). The final alignments were then carried out: unpaired sites were visually determined and removed from the beginning and the end of the resulting matrices. The resulting alignments had lengths of 439 characters.

The data set was analyzed using the Bayesian inference (BI) method implemented in Beast ver. 1.10.1 software (Drummond and Rambaut 2007) to construct phylogeny. For the alignment partition the most appropriate substitution model, shape parameter α and a proportion of invariable sites (pinvar) were estimated using the Bayesian information criterion (BIC) as implemented in jModelTest 2.1.10 (Darriba et al. 2012). This BIC-based model selection procedure TrN+I+G model, α = 0.5130 and pinvar = 0.4620 for 18S rDNA gene. We used the HKY model of nucleotide substitution instead of TrN given that it was the best matching model available for BI. A Yule process tree prior was used as a speciation model. The analysis ran for 5 million generations with chain sampling every 1000 generations. The parameters-estimated convergence, effective sample size (ESS) and burn-in period were checked using the Tracer ver. 1.7.1 software (Drummond and Rambaut 2007). The initial 25% of the trees were removed, the rest were retained to reconstruct a final phylogeny. The phylogenetic tree and posterior probabilities of its branching were obtained on the basis of the remaining trees, having stable estimates of the parameter models of nucleotide substitutions and likelihood. The Maximum Likelihood (ML) analysis was performed using RAxML program (Stamatakis et al. 2008). The nonparametric bootstrap analysis with 1000 replicas was used. The programs FigTree ver. 1.4.4 and Adobe Photoshop CC (19.0) were used for viewing and editing of the trees.

## Results

### Cymbopleura natellia

Taxon classificationPlantaeCymbellalesCymbellaceae

Glushchenko, Kulikovskiy & Kociolek sp. nov.

4BD92B8A-5975-5821-8D81-090636150C67

Figs 1 , 2 , 3 , 4

#### Holotype.

Slide no B209 in collection of MHA, Main Botanical Garden Russian Academy of Science, Moscow, Russia, represented here by Fig. 2J.

#### Reference strain.

Strain B209, isolated in sample No. 11.2.

#### Type locality.

Russia, Eastern Siberia, Buryatia, Zagza River, 52°31.656'N, 107°05.114'E.

#### Description.

**Figure 1.**
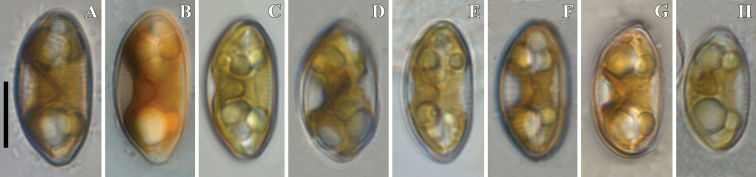
**A–H***Cymbopleuranatellia* Glushchenko, Kulikovskiy & Kociolek, sp. nov. LM, DIC. Strain B209. Size diminution series. Live cells with chloroplast structure. Scale bar: 10 μm.

**Figure 2.**
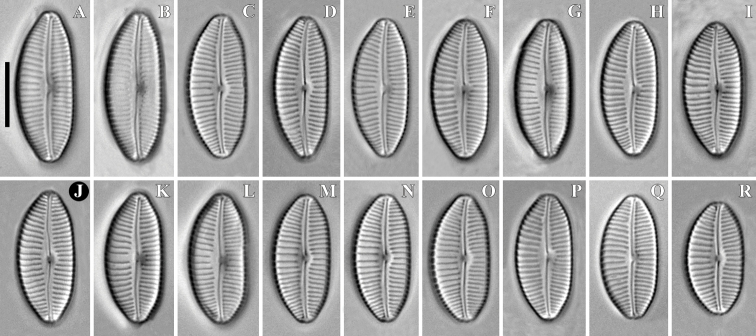
**A–R***Cymbopleuranatellia* Glushchenko, Kulikovskiy & Kociolek, sp. nov. LM, DIC. Strain B209, slide no B209. Size diminution series. Holotype (**J**). Scale bar: 10 μm.

***LM*** (Figs 1 and 2). Cells solitary. A single chloroplast is present per cell. The chloroplast has two lobes, that underlie the valve face, and they are connected by a wide isthmus (Fig. 1). Valves subelliptical, dorsiventral with moderately convex dorsal margin and slightly convex ventral margin, often almost straight near the valve centre. Ends are bluntly rostrate. Length 17.6–23.5 μm (20.5 ± 1.6; n = 30), width 8.7–9.5 μm (9.1 ± 0.2; n = 30). Length-to-width ratio 1.97–2.52. Central area more or less pronounced, rounded, 1/3 to the valve breadth. Axial area narrow, more often weakly expands to central area, less often – almost not expanded beyond the median line of the valve. Raphe filiform near the center, lateral towards the apices, with proximal raphe ends deflected ventrally, tipped with weakly inflated pores. Striae finely punctate, radiate, becoming subparallel and condensing towards to the ends, 13–15 in 10 μm (14 ± 0.7; n = 30) at the central part, 18–20 in 10 μm (19 ± 0.7; n = 30) near the ends. Areolae difficult to resolve in LM (Fig. 2).

**Figure 3.**
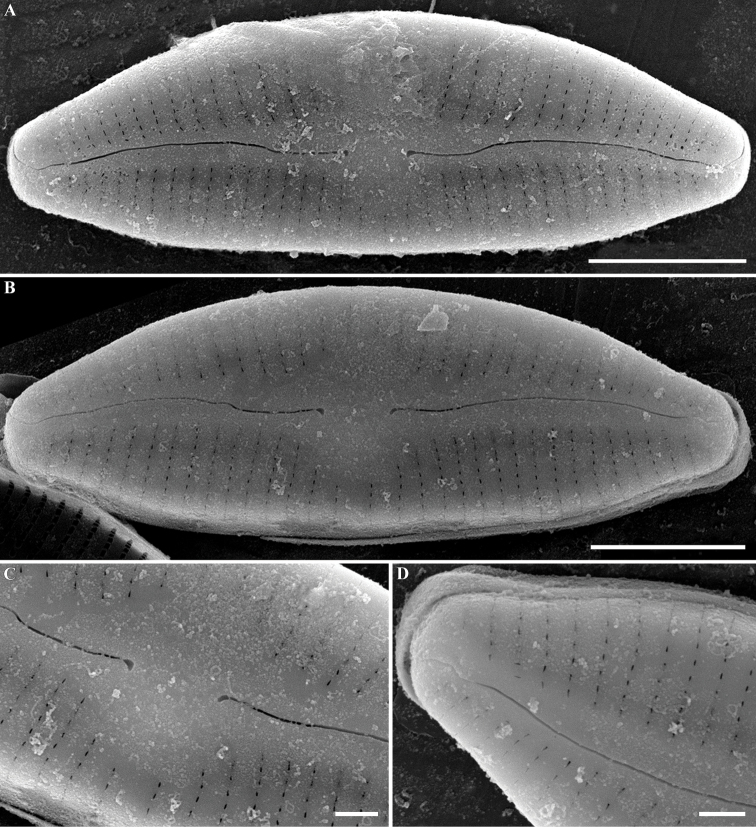
**A–D***Cymbopleuranatellia* Glushchenko, Kulikovskiy & Kociolek, sp. nov. SEM. Strain B209. External view **A, B** the whole valve **C** central area **D** valve end. Scale bars: 5 μm (**A, B**); 1 μm (**C, D**).

***SEM, external view*** (Fig. 3). Valve face is flat. Central area formed by shortened striae. Striae uniseriate, extending to valve face towards mantle on both the dorsal and ventral margins, composed of very small, elongate, lineolate areolae, 30–35 in 10 μm (32.5 ± 1.3; n = 30). Proximal raphe endings are slightly expanded, unilaterally curved. Distal raphe fissures unilaterally curved opposite the proximal raphe ends, extending onto the valve mantle.

**Figure 4.**
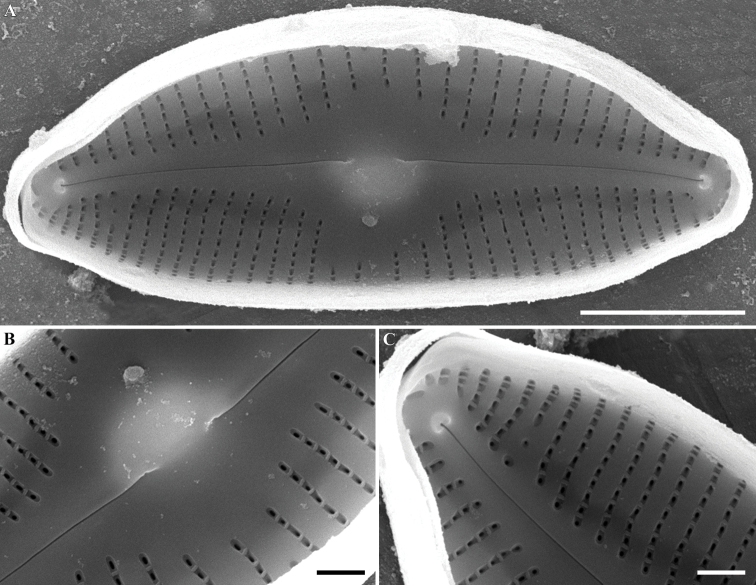
**A–C***Cymbopleuranatellia* Glushchenko, Kulikovskiy & Kociolek, sp. nov. SEM. Strain B209. Internal view **A** the whole valve **B** central area **C** valve end. Scale bars: 5 μm (**A**); 1 μm (**B, C**).

***SEM, internal view*** (Fig. 4). The raphe slits close to proximal endings are arcuate. Proximal raphe endings weakly deflected to the dorsal margin. Distal raphe ends terminated small helictoglossae. Areolae arranged in a series with narrow vimines, compared to the wide interstriae (virgae), occlusions absent but tectullae present.

#### Etymology.

New species is dedicated to our friend Natella Otarovna Gabuadze.

#### Distribution.

As yet known only from the type locality.

#### Sequence data.

Partial 18S rDNA gene sequence comprising V4 domain sequence (GenBank accession number MZ503642).

##### Molecular investigation

**Figure 5.**
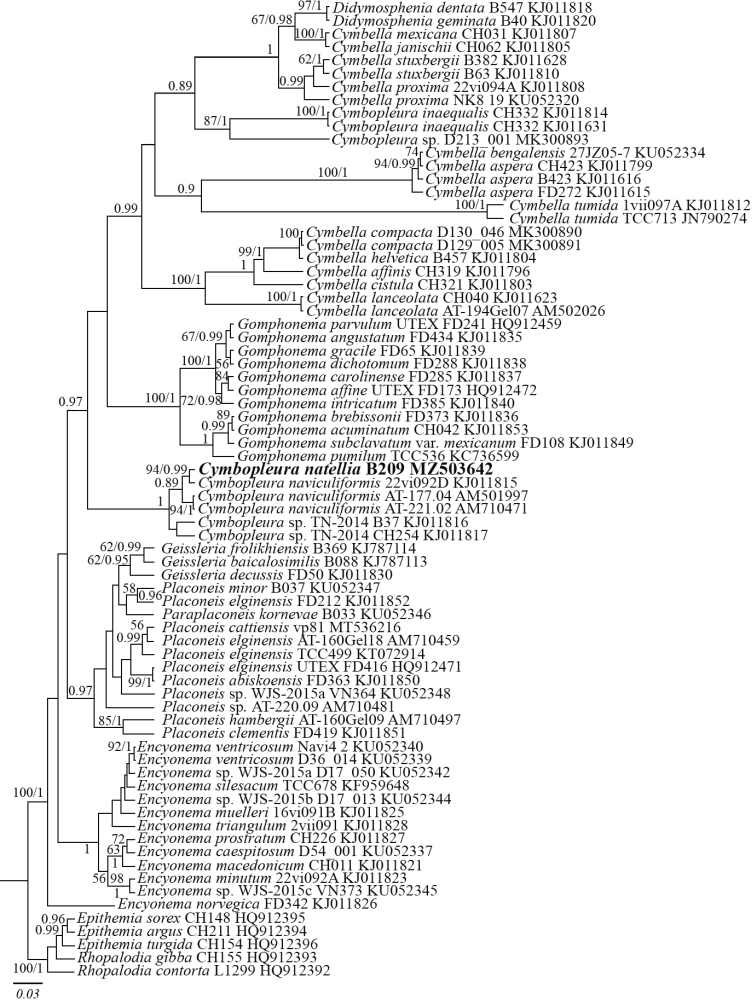
Phylogenetic position of *Cymbopleuranatellia* B209 (indicated in bold) based on Bayesian inference for the partial 18S rDNA gene. Total length of the alignment is 439 characters. Bootstrap supports from ML (constructed by RAxML) and posterior probabilities from BI (constructed by Beast) are presented on the nodes in order. Only BS and PP above 50 and 0.85 are shown. Strain numbers (if available) and GenBank numbers are indicated for all sequences.

The phylogenetic analyses were conducted using a single gene dataset (Fig. 5). Sequences of *Cymbopleura* species as well as *Cymbella*, *Gomphonema*, *Placoneis* and another pennate diatom species were included in the phylogenetic analyses. According to the maximum likelihood (ML) and Bayesian Inference (BI) phylogenetic analyses, *Cymbopleuranatellia* sp. nov. (the strain B209) appeared most closely related to the strain 22 vi092D of *Cymbopleuranaviculiformis* (Auerswald ex Heiberg) Krammer 2003 with high statistical support (ML 94; BI 0.99) and other *Cymbopleura* strains (Fig. 5). We should point out, however, that *Cymbopleura* is non-monophyletic in these analyses, with two strains of *Cymbopleurainaequalis* and an unidentified *Cymbopleura* strain (D213 001 MK300893) having a closer relationship with some *Cymbella* species than *Cymbopleura* strains.

## Discussion

According to the data of molecular analysis, the species *Cymbopleuranatellia* sp. nov. is most closely related to *C.naviculiformis*. According to morphological data, the new species is distinguished by bluntly rostrate, not cuneate valve ends, whereas *C.naviculiformis* has rostrate to subcapitate, narrow-protracted valve ends. The central area of the new species is generally smaller (1/3 of the valve width) than the central area of *C.naviculiformis* (1/2–2/3 of the valve width). The new species differs from *C.naviculiformis* in a shorter valve length (17.6–23.5 µm in the new species versus 26–50 µm in *C.naviculiformis*), as well as, in general, a smaller valve width (8.7–9.5 µm in the new species versus 9.0–13.0 µm in *C.naviculiformis*). The density of striae overlaps in species (13–15 in 10 μm in the central part, 18–20 towards the ends in the new species versus 10–15 in 10 μm in the central part, 16–19 in 10 μm towards the ends in *C.naviculiformis*) and areolae (30–35 in 10 μm in the new species versus 27–35 in *C.naviculiformis*) (Table 1).

**Table 1.** Differences between species of the genus *Cymbopleura*.

	Outline	Ends	Axial area	Central area	Valve length (μm)	Valve width (μm)	Striae in 10 μm	Areolae in 10 μm	Distribution	Reference
*C.natellia* sp. nov.	subelliptical, dorsiventral with moderately convex dorsal margin and slightly convex ventral margin, which more often almost straight near the valve centre	bluntly rostrate, not protracted	narrow, more often weakly expands to central area, less often – almost not expands, almost the median line of the valve	more or less pronounced, rounded, 1/3 the valve breadth	17.6–23.5	8.7–9.5	13–15 at the central part, 18–20 near the ends	30–35	Russia: Baikal Lake	This study
*C.naviculiformis*	elliptic-lanceolate, moderately dorsiventral, dorsal margin strongly arched, ventral margin slightly convex to nearly flat	rostrate to subcapitate, narrow-protracted	narrow, linear or slightly broader towards the relatively large central area, almost in the median line of the valve	rounded, one-half to two-thirds valve width	26–50	9.0–13.0	10–15 at the central part, 16–19 near the ends	27–35	Widely distributed	Krammer 2003; Bahls 2019
*C.designata*	broadly lanceolate, moderately dorsiventral with strongly arched dorsal and ventral margins	apiculate, extending laterally from valve ends	area about 1/4^th^ to 1/5^th^ valve width, slightly curved and narrower near apices	rounded, scarcely wider than the axial area	26–37	9.0–11.6	12–14 at the central part, up to 18 near the ends	26–30	Sub-Arctic and Arctic	Lange-Bertalot and Genkal 1999; Bahls 2019
*C.frequens*	not or very slightly dorsiventral, subelliptical-lanceolate, dorsal an ventral margins moderately arched	protracted, apiculate to rostrate	narrow, linear, narrowing slightly towards to the ends, almost median line of the valve	irregular, asymmetric space of different extent	14–38	6–9	11–14 at the central part, 16 near the ends	30–36	Holarctic	Krammer 2003; Kulikovskiy et al. 2016; Bahls 2019
*C.hercynica*	slightly dorsiventral, broadly subelliptical to elliptical-lanceolate, dorsal margins strongly, ventral margin slightly arched	apiculate to subrostrate protracted	very narrow, almost the median line of the valve	1/3–1/2 the valve breadth, distinctly set off, rounded, sometimes asymmetrical and then more well-developed dorsally	16–40	7–10	12–15 at the central part, up to 20 near the ends	32–36	Holarctic, saline habitats or with higher electrolyte content	Krammer 2003; Li et al. 2007

*Cymbopleuranatellia* sp. nov. is morphologically similar to *Cymbopleuradesignata* (Krammer) Bahls (in Bahls 2019). The two species are close in terms of density of striae (13–15 in the central part, 18–20 at the ends in the new species versus 13–15 in the central part, 18–20 at the ends in *C.designata*). The main difference between these two species is the shape of valves. The new species has subelliptical valve outlines while *C.designata* has a wide lanceolate shape of the valves. The central area of the new species is more differentiated than that of *C.designata*, the central area of which is slightly wider than the axial area. The ends of *C.designata* are apiculate, extending laterally from valve ends. There is also a difference in areolae density (30–35 in 10 µm in the new species versus 26–30 in 10 µm in *C.designata*).

*Cymbopleuranatellia* sp. nov. is also morphologically similar to *C.frequens*Krammer 2003. These two species have a similar number of areolae (30–35 in 10 μm in the new species and 30–36 in 10 μm in *C.frequens*). At the same time, the species differ in the outline of the valves, with the valves of *C.frequens* being almost naviculoid or slightly dorsiventral, while in *C.natellia* sp. nov. the valves are subelliptic and evidently dorsiventral. The ends of *C.frequens* are protracted, apiculate to rostrate.

*Cymbopleurahercynica* (A.W.F. Schmidt) Krammer 2003 is another species that is morphologically similar to the new species, in terms of stria density (12–15 in the central part, up to 20 in 10 μm toward to the ends in *C.hercynica* versus 13–15 in the central part, 18–20 in 10 μm to the ends in the new species) and density of areolae (32–36 in 10 μm in *C.hercynica* versus 30–35 in 10 μm in the new species). Also, the two species are generally similar in valve shape. The species differ in the shape of the valve ends, in the new species the ends are wide, in *C.hercynica* they are apiculate to subrostrate protracted. The width of the central area of *C.hercynica* reaches 1/2 of the total valve width; in the new species, this ratio does not exceed 1/3. It should be noted that the new species was isolated from the Zagza River, where the water conductivity is low, while *C.hercynica* prefers brackish-water habitats or reservoirs with increased salinity (Krammer 2003; Li et al. 2007).

## Supplementary Material

XML Treatment for Cymbopleura natellia

## References

[B1] Bahls L (2019) Diatoms from Western North America 2. *Cymbellafalsa*, *Cymbopleura and Delicatophycus* (Bacillariophyta) – taxonomy, ecology, biogeography. Helena, Montana: published by the author, 114 pp.

[B2] Darriba D Taboada GL Doallo R Posada D (2012) jModelTest 2: More models, new heuristics and parallel computing. Nature Methods 9(8): e772. 10.1038/nmeth.2109 PMC4594756 22847109

[B3] Drummond AJ Rambaut A (2007) BEAST: Bayesian evolutionary analysis by sampling trees. BMC Evolutionary Biology 7(1): e214. 10.1186/1471-2148-7-214 PMC2247476 17996036

[B4] Glushchenko AM Kuznetsova IV Kociolek JP Kulikovskiy MS (2019) *Karthickiaverestigmata* gen. et sp. nov. - an interesting diatom with frustular morphology similar to several different cymbelloid diatom genera. Phycologia 58(6): 605–613. 10.1080/00318884.2019.1626605

[B5] Guillard RRL Lorenzen CJ (1972) Yellow-green algae with chlorophyllide c. Journal of Phycology 8: 10–14. 10.1111/j.1529-8817.1972.tb03995.x

[B6] Katoh K Toh H (2010) Parallelization of the MAFFT multiple sequence alignment program. Bioinformatics (Oxford, England) 26(15): 1899–1900. 10.1093/bioinformatics/btq224 PMC2905546 20427515

[B7] Krammer K (1982) Valve morphology and taxonomy in the genus *Cymbella* C.A. Agardh. Morphology of Diatom Valves 11: 1–299.

[B8] Krammer K (2003) *Cymbopleura*, *Delicata*, *Navicymbula*, *Gomphocymbellopsis*, *Afrocymbella*. Diatoms of Europe 4: 1–530.

[B9] Kulikovskiy MS Lange-Bertalot H Metzeltin D Witkowski A (2012) Lake Baikal: Hotspot of endemic diatoms I. Iconographia Diatomologica 23: 7–607.

[B10] Kulikovskiy MS Glushchenko AM Genkal SI Kuznetsova IV (2016) Identification book of diatoms from Russia. Yaroslavl, Filigran, 804 pp.

[B11] Kumar S Stecher G Tamura K (2016) MEGA7: Molecular Evolutionary Genetics Analysis Version 7.0 for Bigger Datasets. Molecular Biology and Evolution 33(7): 1870–1874. 10.1093/molbev/msw054 27004904 PMC8210823

[B12] Lange-Bertalot H Genkal SI (1999) Diatoms from Siberia I. Islands in the Arctic Ocean (Yugorsky-Shar Strait). Iconographia Diatomologica 6: 1–292.

[B13] Li Y Gong Z Xie P Shen J (2007) Floral survey of the diatom genera *Cymbella* and *Gomphonema* (Cymbellales, Bacillariophyta) from the Jolmolungma Mountain Region of China. Cryptogamie. Algologie 28(3): 209–244.

[B14] Pomazkina GV Rodionova EV (2014) Diatoms of the family Cymbellaceae of Lake Baikal. Atlas and key. Novosibirsk, Nauka, 242 pp.

[B15] Round FE Crawford RM Mann DG (1990) The Diatoms. Biology and Morphology of the Genera. Cambridge University Press, Cambridge, 747 pp.

[B16] Stamatakis A Hoover P Rougemont J (2008) A rapid bootstrap algorithm for the RAxML web-servers. Systematic Biology 75(5): 758–771. 10.1080/10635150802429642 18853362

[B17] Zimmermann J Jahn R Gemeinholzer B (2011) Barcoding diatoms: Evaluation of the V4 subregion on the 18S rRNA gene, including new primers and protocols. Organisms, Diversity & Evolution 11(3): 173–192. 10.1007/s13127-011-0050-6

